# Electromyographic Analysis of Latissimus Dorsi Activation During Common Resistance Training Exercises: A Narrative Review

**DOI:** 10.3390/jfmk11030315

**Published:** 2026-08-12

**Authors:** Domenico Di Fonza, Gloria Di Claudio, Gianmaria Colantuono, Lorenzo Persichini, Nicola Cerulo, Benedetta Sangregorio, Andrea Buonsenso, Giovanni Fiorilli, Giuseppe Calcagno, Alessandra di Cagno

**Affiliations:** 1 Department of Medicine and Health Sciences, University of Molise, 86100 Campobasso, Italy; difonzad@gmail.com (D.D.F.); gloria.diclaudio@univr.it (G.D.C.); gimmy.colantuono@gmail.com (G.C.); n.cerulo@studenti.unimol.it (N.C.); sangregoriobenedetta@gmail.com (B.S.); giuseppe.calcagno@unimol.it (G.C.); 2 Department of Neurosciences, Biomedicine and Movement, University of Verona, 37129 Verona, Italy; 3 Department of Movement, Human and Health Sciences, University of Rome “Foro Italico”, 00135 Rome, Italy; l.persichini1@studenti.uniroma4.it (L.P.); alessandra.dicagno@uniroma4.it (A.d.C.); 4 Department of Theoretical and Applied Sciences, eCampus University, 22060 Novedrate, Italy; andrea.buonsenso@uniecampus.it; 5 Department of Human Sciences, Guglielmo Marconi University, 00193 Rome, Italy

**Keywords:** latissimus dorsi, electromyography, resistance training, muscle activation, pulling exercises

## Abstract

**Background**: The latissimus dorsi (LD) is a primary muscle involved in pulling exercises used in resistance training, contributing to shoulder extension, adduction and internal rotation, with relevance to athletic performance, injury prevention and rehabilitation as well as to upper-body aesthetics. **Aim**: to identify which exercise configurations are most consistently associated with greater latissimus dorsi electromyographic (EMG) amplitude. **Methods**: This SANRA-informed narrative review synthesized surface and high-density EMG evidence across major pulling exercises, for the lat pull-down, pullover, seated row, barbell row, bent-over row and pull-up. Twenty-three eligible studies were included and appraised using a six-criterion methodological checklist. **Results**: In the lat pull-down, an anterior bar path was the configuration most consistently associated with greater LD EMG amplitude, whereas the effects of grip width were inconsistent. In the seated row, a narrower grip and a lower shoulder-abduction angle were each associated with greater LD amplitude in separate studies; these two variables have not been tested in combination and should not be read as a single validated configuration. Among rowing variants, the inverted row produced the highest LD amplitude in a single, small preliminary study that requires replication. Pull-up variations showed broadly similar LD activation across grip configurations, while the pullover appeared to preferentially activate the pectoralis major rather than selectively target the LD. **Conclusions**: Current evidence tentatively supports the anterior lat pull-down and, within the seated row, a narrower grip and a lower shoulder-abduction angle (evaluated independently) as the configurations most consistently associated with greater LD EMG amplitude; these findings reflect acute EMG amplitude rather than longitudinal training outcomes and should be treated as an evidence-informed starting point rather than a definitive exercise prescription. Across pulling exercises, the humerus-to-trunk relationship may be a key biomechanical determinant of muscular load distribution.

## 1. Introduction

The latissimus dorsi muscle represents one of the main muscle groups involved in pulling exercises used in the context of strength and conditioning and resistance training [1]. From an aesthetic point of view, the latissimus dorsi is strongly associated with back width and the characteristic “V-shaped” trunk appearance, which is why its development is frequently sought both by athletes and by individuals who practice resistance training for aesthetic purposes. At the same time, from a functional perspective, the latissimus dorsi contributes to fundamental movements of the upper limb, including extension, adduction and internal rotation of the humerus, making it essential in numerous athletic and sport-specific movements [2].

Beyond aesthetics and general athletic performance, latissimus dorsi function has direct relevance to injury prevention, rehabilitation, and clinical practice. As a large thoracohumeral muscle crossing the glenohumeral joint, the latissimus dorsi contributes to the muscular control of humeral head translation: biomechanical work has shown that thoracohumeral muscle activity, including that of the latissimus dorsi, alters glenohumeral kinematics and joint reaction forces during active abduction [3]. Coordinated activity of the scapulothoracic and scapulohumeral musculature is central to normal scapulohumeral rhythm, and altered scapular position and motion are associated with a range of shoulder pathologies [4,5]. In throwing and other overhead sports, the latissimus dorsi is among the muscles most strongly recruited during the acceleration phase of the movement and contributes to deceleration of the upper limb [6], and exercises addressing the glenohumeral and scapulothoracic musculature are a recurring component of shoulder rehabilitation and prevention programs [7]. Evidence-based exercise selection for this muscle therefore has practical relevance not only for hypertrophy- or performance-oriented resistance training, but also for injury-prevention and rehabilitation programming, which reinforces the applied value of synthesizing the available EMG literature.

For this reason, the scientific literature has progressively directed increasing attention toward the analysis of the most commonly used exercises for latissimus dorsi training, with the aim of understanding which movements are most consistently associated with greater acute neuromuscular activation, as measured by EMG amplitude [1,8,9,10]. Among the most frequently studied exercises are the lat pull-down in all its variations, the pull-over, the seated row with different working angles, grips and hand positions, the barbell row, bent-over row and various pull-up variations [10].

Most studies in the literature use surface electromyography (sEMG) [11] or high-density electromyography (HD-sEMG) [12] to assess the degree of muscle activation during the execution of dynamic exercises. These methods allow for the analysis of which muscle groups are most involved during movement and the quantification of their relative EMG amplitude, generally expressed as a percentage of maximal voluntary isometric contraction (%MVIC) [13]. It is important to note at the outset that EMG amplitude is an indirect proxy for the level of neural drive to a muscle during a given task, and does not, on its own, establish the training effectiveness of an exercise for hypertrophy, strength development, or long-term functional adaptation; this distinction is discussed further in Section 4 (Discussion) and revisited throughout the review.

One of the most debated aspects in the literature concerns the ability of an exercise to preferentially recruit (“isolate”, in the terminology used by some of the reviewed sources) the latissimus dorsi compared to the other muscles involved in different exercises [1,8,9]. The term “isolation” is used cautiously throughout this review: because the exercises considered are compound, multi-joint movements, no pulling exercise engages the latissimus dorsi to the complete exclusion of synergistic musculature, and apparent differences in relative muscle contribution are more accurately described in terms of EMG amplitude or muscle excitation than true anatomical isolation. Although many exercises show high levels of LD EMG amplitude, different technical variations, including grip width, hand orientation, movement trajectory, trunk inclination and the use of different equipment, can modify [14,15] significantly or not [1,9] the pattern of muscle EMG amplitude across the muscles involved. Understanding these differences is relevant both for performance optimization and for programming training oriented toward muscle hypertrophy, although the acute EMG evidence reviewed here cannot on its own confirm hypertrophic outcomes.

Moreover, the interpretation of electromyographic data presents numerous methodological issues that deserve attention. Differences in experimental protocols, EMG signal normalization, electrode placement, relative load intensity used, and subjects’ training experience can significantly influence the results reported by studies [13]. Consequently, it is necessary to critically analyze the available literature in order to identify possible convergences or discrepancies among different scientific works, and to avoid direct cross-study ranking of exercises when protocols and normalization procedures are not comparable.

Therefore, the aim of this narrative review is to analyze the scientific literature related to the most commonly used exercises for latissimus dorsi training, evaluating which exercises and technical variations are most consistently associated with higher levels of LD EMG amplitude. At the same time, the EMG amplitude of other synergistic muscle groups will be considered, with particular attention to the relative muscle contribution of the latissimus dorsi during different pulling exercises. The analysis of the available evidence could provide useful, hypothesis-generating indications for strength and conditioning professionals, athletic trainers and resistance training practitioners in the selection of exercises according to training objectives, while explicitly acknowledging that acute EMG data cannot substitute for longitudinal training studies when making causal claims about hypertrophic or strength outcomes.

## 2. Materials and Methods

### 2.1. Study Design and Rationale

A narrative review design was selected because the available literature on latissimus dorsi EMG activity during pulling exercises is characterized by substantial heterogeneity in relative load, repetition scheme, movement velocity, fatigue level, range of motion, contraction type, electrode placement, signal processing, and normalization procedures (detailed further in Section 4), which precludes meaningful quantitative pooling of effect sizes in a systematic review or meta-analysis. A narrative synthesis was judged more appropriate to integrate exercise-specific biomechanical patterns across heterogeneous outcome metrics (e.g., %MVIC, normalized RMS EMG, HD-sEMG excitation maps) while allowing explicit, study-by-study discussion of methodological limitations.

At the same time, several elements of this review depart from a purely descriptive narrative format and are more characteristic of a structured evidence synthesis: a database search using an explicit Boolean strategy, predefined eligibility criteria applied through a documented screening process, and a systematic (rather than selective) presentation of study-level methodological characteristics. To reconcile these two aspects, the review was guided by SANRA (Scale for the Assessment of Narrative Review Articles) framework [16] for the overall structure and interpretive approach, while selected reporting elements of PRISMA 2020—namely a structured search strategy, predefined eligibility criteria, and a study-selection flow description (Section 2.4)—were incorporated to improve transparency and reproducibility. This hybrid approach was preferred over a full systematic review because the quantitative pooling step (meta-analysis) that would typically follow a PRISMA-compliant systematic search is not statistically justified given the outcome and protocol heterogeneity described above; the review therefore remains narrative in its synthesis and interpretation, while adopting the more transparent, reproducible search-and-selection reporting associated with systematic approaches.

Consistent with its exercise-focused objective, the evidence synthesis (Section 3, Results) is organized by exercise category (lat pull-down, pullover, seated row, barbell row, bent-over row, pull-ups) rather than by biomechanical variable or by study. This organizing structure was chosen because it mirrors how strength and conditioning professionals select among exercises in practice and allows methodological characteristics and findings to be compared directly within a given movement pattern, where protocols are more likely to be comparable; cross-exercise-family comparisons are made only where explicitly justified and are otherwise avoided, for the reasons detailed in Section 4.

### 2.2. Literature Search Strategy

A literature search was conducted across PubMed/MEDLINE, Scopus, and Web of Science (Core Collection), covering the period from November 2002 to March 2026. The starting date corresponds to the publication of the earliest study meeting the eligibility criteria identified during the search [15], which represents the earliest EMG investigation of lat pull-down hand-position variants retrieved. The final search was completed on 10 March 2026. The search was supplemented by a manual screening of Google Scholar, restricted to the first 200 results ranked by relevance and screened by title (and, when necessary, abstract), and by backward and forward citation screening of the reference lists of all included articles. No language restriction was applied a priori; in practice, all studies meeting the eligibility criteria were published in English.

The search string, adapted to the syntax of each database, combined the following concept blocks with the Boolean operator AND, with terms within each block combined with OR:

Block 1 (muscle): “latissimus dorsi” Block 2 (methodology): “electromyography” OR “EMG” OR “surface electromyography” OR “sEMG” OR “high-density electromyography” OR “HD-sEMG” Block 3 (exercise): “lat pull-down” OR “lat pulldown” OR “pull-down” OR “seated row” OR “cable row” OR “barbell row” OR “bent-over row” OR “bent over row” OR “pull-over” OR “pullover” OR “pull-up” OR “pull up” OR “chin-up” OR “chin up” OR “muscle activation” OR “resistance training” OR “strength training”.

In PubMed, the search was restricted to the Title/Abstract fields ([tiab]); in Scopus, to TITLE-ABS-KEY; in Web of Science, to Topic (TS=). No publication-type filter was applied at the database level; publication type was instead assessed during full-text screening (Section 2.3).

### 2.3. Eligibility Criteria and Study Selection

Eligible records were original experimental or observational studies, published in peer-reviewed journals or as full conference abstracts containing sufficient methodological detail (sample size, exercise protocol, and EMG methodology) to permit critical appraisal, reporting surface or high-density surface EMG amplitude of the latissimus dorsi during a dynamic (concentric/eccentric) or isometric pulling or pullover exercise commonly prescribed in resistance training. Eligible participants were healthy adults, whether untrained, recreationally active, or resistance-trained. Studies conducted exclusively in pediatric, elderly, or clinical/rehabilitation populations, cadaveric or musculoskeletal-modeling studies without EMG data, reviews, meta-analyses, editorials, and non-original commentaries were excluded. One conference abstract [17] met these criteria despite not reporting its sample size or EMG normalization procedure; consistent with its abstract-only format and low methodological-quality score, its findings are explicitly identified as preliminary throughout the text and tables and do not, on their own, support any practical recommendation (Section 2.5). Nevertheless, its inclusion was considered informative within the context of this review, as it contributes preliminary evidence to the overall body of literature and may help inform the interpretation of the available evidence, while its methodological limitations and the resulting uncertainty are explicitly acknowledged.

The search results were independently screened according to titles, abstracts, and full text by three researchers. Subsequently, the same three researchers independently extracted the characteristics and findings of the included studies into a standardized extraction form; extracted entries were then cross-checked by all three researchers against the original source to verify accuracy. In cases of disagreement, whether at the screening or data-extraction stage, a fourth researcher was consulted to resolve the discrepancy.

### 2.4. Study Selection Flow

**Figure 1:**
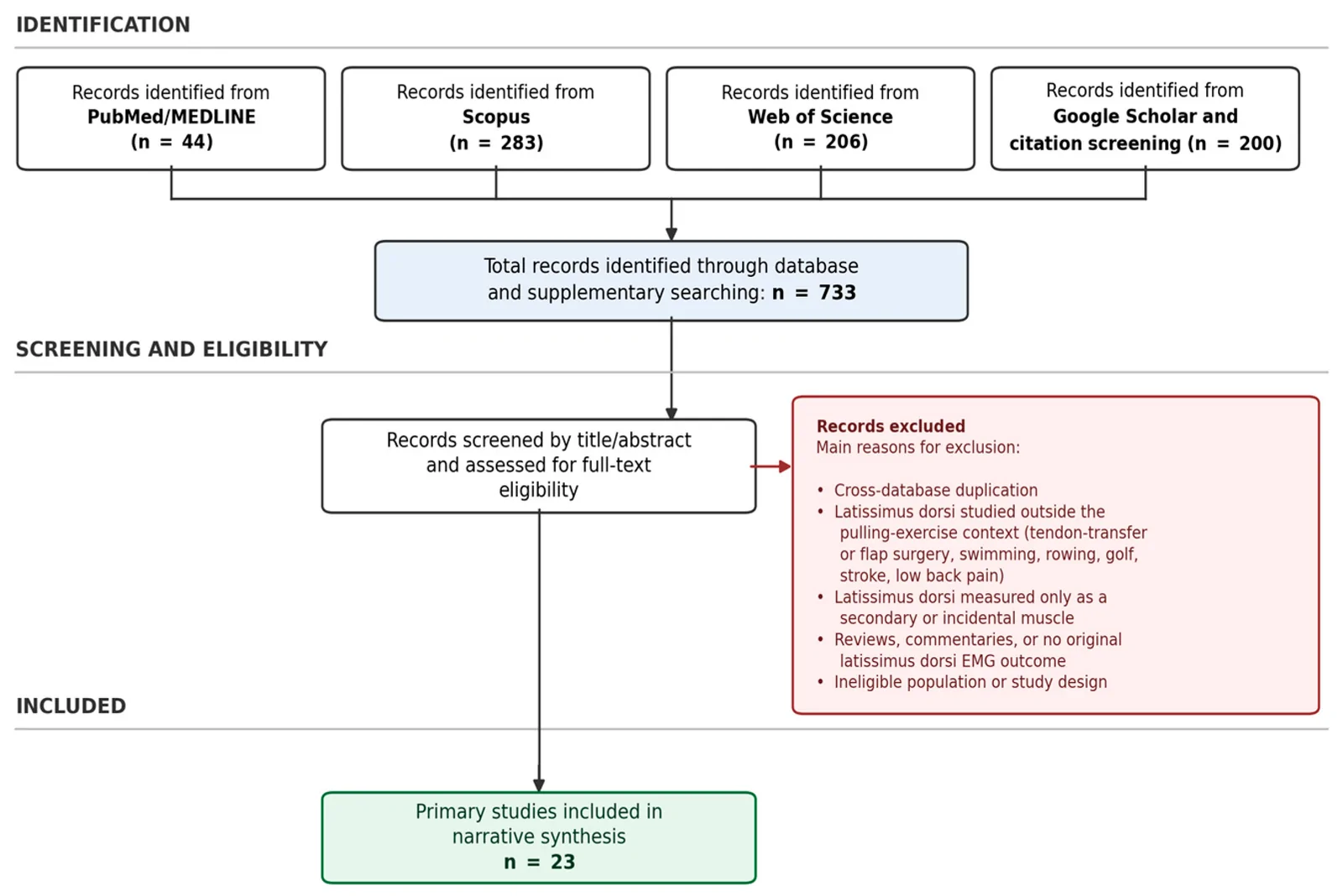
PRISMA 2020-style flow diagram of the study-selection process, from the records identified through database and supplementary searching to the 23 primary studies included in the narrative synthesis. The main reasons for exclusion at the title/abstract and full-text stages are summarized in the right-hand box.

Following the structure recommended by PRISMA 2020 for reporting the study-selection process, 23 primary experimental studies met the eligibility criteria and constitute the evidentiary basis of this narrative review (Table 1; references [1,8,9,10,14,15,17,18,19,20,21,22,23,24,25,26,27,28,29,30,31,32,33]). The remaining 14 references were not subject to the eligibility screening described in Section 2.3 and are used exclusively for background and interpretive purposes: 5 references providing anatomical or general EMG-methodological background ([2,11,12,13,34]: latissimus dorsi anatomy, EMG modeling, HD-sEMG technique, %MVIC normalization, and general seated-row technique guidance); 4 references underpinning the reporting and appraisal frameworks used in this review (SANRA [16]; the Downs & Black checklist [35]; SENIAM electrode-placement recommendations [36]; and a general strength and conditioning reference [37]); and 5 clinical and sports-medicine references supporting the rationale outlined in Section 1 [3,4,5,6,7]. The reference list therefore totals 37 entries (23 primary + 14 background/methodological), consistent with the numbering in the References section. The study-selection process is summarized in Figure 1.

The search retrieved 44 unique records in PubMed/MEDLINE, 283 records in Scopus and 206 records in Web of Science Core Collection; together with the 200 Google Scholar records screened by title and the records identified through backward and forward citation screening, this yielded 733 records identified before de-duplication across sources. This total reflects the substantial overlap expected across three large, partially redundant bibliographic databases combined with a deliberately broad exercise block, which intentionally included generic terms such as “muscle activation” and “resistance training” to maximize sensitivity. Records were excluded at the title/abstract stage principally because they were cross-database duplicates; because the latissimus dorsi was studied outside the resistance-training pulling context (for example in tendon-transfer or myocutaneous-flap surgery, or during swimming, competitive rowing, golf, or in post-stroke and low-back-pain populations); or because the latissimus dorsi was measured only as a secondary or incidental muscle in studies of other exercises or muscle groups. The full texts of the remaining records were assessed against the criteria set out in Section 2.3, and 23 studies were included in the synthesis.

Screening was performed as described in Section 2.3, with three authors screening independently and a fourth author resolving disagreements. The review was not based on a study list defined in advance of the search: the evidence base was assembled through database searching followed by backward and forward citation screening and was subsequently verified against the eligibility criteria specified in Section 2.3. No review protocol was registered prospectively.

### 2.5. Data Synthesis and Quality Considerations

Given the narrative nature of the review, no single validated clinical risk-of-bias instrument (e.g., the Cochrane Risk-of-Bias tool, developed for randomized controlled trials, or the PEDro Scale, developed for physiotherapy intervention trials) was applied, as neither is designed for acute, within-subject biomechanical/EMG crossover studies of the kind that make up this evidence base (single-session comparisons without a clinical intervention, follow-up, or patient-relevant outcome). Instead, each included study was appraised using a transparent, review-specific six-criterion checklist informed by general methodological-quality domains described by Downs and Black [35] and by SENIAM electrode-placement and normalization recommendations [36], adapted to the acute EMG literature: (1) sample size ≥ 12 participants; (2) both sex and training status of participants reported; (3) exercise load standardized relative to individual capacity (e.g., RM, %1RM, %body weight) or, for isometric protocols, a standardized maximal-effort procedure; (4) EMG normalization procedure explicitly reported (%MVIC or an equivalent normalized excitation measure); (5) dynamic (rather than isometric-only) assessment; and (6) use of high-density surface EMG (providing greater spatial resolution than conventional bipolar sEMG). Each criterion was scored 1 (met) or 0 (not met/not reported), yielding a summary indicator from 0–6 for each study (Table 2). This indicator is descriptive and review-specific rather than a validated scale, and is intended to make transparent, at a glance, which observations in Section 3 and Section 4 rest on more versus less methodologically robust sources; it does not substitute for a full risk-of-bias judgment in the systematic-review sense.

Studies with small samples (fewer than approximately 10–15 participants), single-sex or narrowly defined cohorts, or preliminary/abstract-only designs are explicitly identified as such in Table 1 and Table 2, and their findings are framed with corresponding caution in the narrative text rather than presented as generalizable conclusions. Evidence derived from a single small study, or from a conference abstract, is not treated as equivalent in strength to a converging pattern observed across multiple independent, adequately powered, peer-reviewed studies; where this distinction is relevant it is made explicit in Section 3 and Section 4.

Throughout this review, the terms “EMG amplitude” and “muscle excitation” are used to describe the measured outcome (normalized EMG signal amplitude, expressed as %MVIC or an equivalent normalized unit), rather than “activation” in a general physiological sense or “isolation” in an anatomical sense. Statements regarding differences between conditions reflect statistical significance as reported by the original authors; where the primary studies did not report effect sizes or confidence intervals, this is noted, and no independent judgment of clinical or practical meaningfulness is imposed beyond what the original authors reported, since no consensus threshold for a minimally important difference in EMG amplitude currently exists in this literature. Where a primary study did report standardized effect sizes, these are reproduced in Table 1 and in the corresponding text of Section 3; in the present evidence base this was the case for two studies only (Padovan et al., 2025 [9]; Fischer et al., 2025 [27]).

## 3. Results

Consistent with the exercise-based synthesis structure described in Section 2.1, findings are presented below by exercise category. Methodological characteristics of each included study, including EMG normalization procedure and electrode/acquisition approach (conventional bipolar sEMG vs. HD-sEMG), are summarized in Table 1, and a transparent methodological-quality indicator for each study is reported in Table 2 (Section 2.5). Regarding electrode placement, the majority of the conventional-sEMG studies included here report bipolar electrode positioning broadly consistent with SENIAM (Surface Electromyography for the Non-Invasive Assessment of Muscles) recommendations [36] for the latissimus dorsi (typically over the muscle belly, inferolateral to the inferior angle of the scapula, along the presumed fiber direction), although exact inter-electrode distance, precise anatomical landmarks and skin-preparation procedures are not uniformly reported across the included studies; this is noted as a source of between-study heterogeneity in Section 4. EMG acquisition systems (hardware/software) also differ across studies and are generally specific to each laboratory; where reported, this is reflected in the “EMG Normalization/Electrodes” column of Table 1. The three high-density surface EMG (HD-sEMG) studies included [9,20,26] used a fundamentally different, grid-based acquisition approach, which provides greater spatial resolution but is not directly comparable to single-site conventional sEMG in absolute terms; comparisons between conventional and HD-sEMG findings are therefore made cautiously throughout this section and are revisited in Section 4.

### 3.1. Lat Pull-Down

The lat pull-down is one of the most widely used pulling exercises in strength training and hypertrophy programs targeting the latissimus dorsi, and its widespread adoption has prompted a substantial body of research aimed at identifying which execution variant is associated with greater target-muscle EMG amplitude [1]. This exercise family has the largest evidence base of those reviewed here (six primary studies identified, combined *n* ≈ 115). The primary variables investigated include grip width, forearm orientation (pronated vs. supinated grip), and pull direction (anterior vs. posterior to the head), often examined in combination.

Regarding grip width, the available evidence is inconsistent. An early study in ten healthy men (Signorile et al., 2002 [15]) reported that a wide anterior grip produced greater LD EMG amplitude than narrow and supinated variants, in both the concentric and eccentric phases. This difference was not confirmed in later, larger studies: Andersen et al. (2014 [18], *n* = 15 men) found no significant differences in LD EMG amplitude between narrow, medium and wide grips, and the same absence of effect across grip width and additional execution variants was reported by Buonsenso et al. (2025 [1], *n* = 40 resistance-trained men). On the balance of the current evidence, grip width is not sufficiently supported as a determinant of LD EMG amplitude in this exercise, and the isolated positive finding from the smallest and earliest of these studies is not a sufficient basis for a general recommendation.

With respect to forearm orientation, a pronated grip was associated with greater LD EMG amplitude than a supinated grip in a study of 12 healthy men (Lusk et al., 2010 [14]), whereas the supinated variant, despite eliciting lower LD EMG amplitude, was associated with greater biceps brachii EMG amplitude. This aspect is potentially relevant for training program design: a medium or narrow grip, irrespective of forearm orientation, was associated with higher biceps brachii EMG amplitude in the Andersen et al. (2014 [18]) sample, making these variants a plausible option when greater elbow-flexor involvement is a training goal.

The comparison between anterior and posterior pull direction represents one of the most debated issues in the literature. In a study of 24 trained men, the anterior execution displayed a more distributed EMG amplitude profile among the primary muscles involved, with greater LD and pectoralis major EMG amplitude during the concentric phase, compared with the posterior (behind-the-neck) variant (Sperandei et al., 2009 [19]). The posterior variant, by contrast, was associated with greater posterior deltoid and biceps brachii EMG amplitude. A separate high-density EMG comparison in 14 resistance-trained men found the effect to be phase-dependent: the anterior variant produced greater latissimus dorsi and pectoralis major excitation during the descending (concentric) phase, whereas the posterior variant produced greater latissimus dorsi excitation during the ascending (eccentric) phase (Padovan et al., 2024 [20]). Neither of these studies directly assessed joint symptoms, injuries, or prospective adverse outcomes; the frequently cited concern regarding elevated cervical-spine and shoulder-joint stress during the behind-the-neck variant is a biomechanical consideration grounded in the extreme end-range shoulder external rotation and abduction the movement requires, rather than a demonstrated difference in injury risk from the EMG literature reviewed here. On this basis, the anterior execution can be considered a reasonable default for most practitioners on biomechanical grounds, while acknowledging that direct evidence of superior safety, rather than plausible biomechanical rationale, is currently lacking.

An additional execution parameter concerns trunk inclination: in a study of 40 resistance-trained men with at least five years of training experience, performing the exercise at 70% 1RM (Buonsenso et al., 2025 [1]), a slight posterior lean of approximately 30° combined with a wide pronated grip was the only condition found to produce a significant increase in posterior deltoid EMG amplitude compared with the other variants, without altering LD EMG amplitude. While this finding may be of interest when greater posterior shoulder musculature involvement is sought, it does not modify the principal observations for the exercise.

In summary, the available literature most consistently supports the anterior pull direction as the configuration of choice in the lat pull-down, on the basis of a more distributed EMG amplitude profile among the primary muscles involved and a more conservative shoulder and cervical position. Grip width, by contrast, has not produced a consistent effect on LD EMG amplitude across studies and is therefore not presented here as a determinant of latissimus dorsi EMG amplitude; where the training objective is greater elbow-flexor involvement, a medium or narrow grip remains a plausible option on the basis of the biceps brachii data. The posterior-to-head variant does not show a consistent LD EMG amplitude advantage over the anterior variant and is associated with a less favorable biomechanical loading profile at the cervical spine and shoulder joint on theoretical grounds; in the absence of direct injury-outcome data, this should be framed as a biomechanical caution rather than a demonstrated safety difference. It should also be noted that the reviewed studies present limitations related to relatively small sample sizes (*n* = 10–40, predominantly or exclusively male) and the predominant use of conventional surface electromyography, which does not capture the spatial distribution of muscle activation with the same precision as HD-sEMG [20]. This warrants caution in generalizing the conclusions to female, specialized athletic, or high-performance populations, which were not represented in the reviewed samples.

### 3.2. Pull-Over

The pullover is a resistance exercise characterized by its capacity to simultaneously engage the latissimus dorsi and the pectoralis major, making it a relatively unique movement within pulling-exercise taxonomy [21]. Despite this distinctive feature, the body of evidence examining its neuromuscular demands remains comparatively limited (three primary studies identified, combined *n* ≈ 43), and the available studies point to a strong dependence of relative muscle EMG amplitude on shoulder joint position and specific execution conditions.

Under traditional execution conditions, in a study of eight healthy male volunteers, the pullover was associated with greater pectoralis major than latissimus dorsi EMG amplitude, more consistent with an anterior-chain-dominant than a lat-dominant pattern of EMG amplitude (Marchetti & Uchida, 2011 [21]). This finding, from a small single-sex sample, challenges the common assumption that the pullover functions primarily as a lat-dominant exercise, although replication in larger and more diverse samples is warranted before this is treated as an established pattern. The degree to which each muscle shows EMG amplitude does not appear, in a study of 15 recreationally trained men, to be determined primarily by the choice of exercise itself, but rather by shoulder joint angle: across a range of positions from 0° to 180° under isometric testing conditions, the LD and posterior deltoid displayed EMG amplitude patterns broadly comparable between the pullover and the pulldown, while pectoralis major EMG amplitude proved more sensitive to joint position than to exercise selection (Teixeira et al., 2022 [8]). Because this comparison was obtained under isometric conditions in a small, single-sex sample, its transferability to dynamic training contexts should be considered provisional.

When comparing the barbell pullover with the straight-arm pulldown across different grip widths in a sample of 20 healthy, physically active adults, the pullover generally produced higher overall EMG amplitude for most muscles studied, with the notable exception of the latissimus dorsi and triceps brachii, for which no such increase was observed (Muyor et al., 2022 [22]). This pattern further reinforces the interpretation of the pullover as a movement characterized by distributed EMG amplitude across several regions, rather than a highly selective latissimus dorsi exercise. The straight-arm pulldown, by contrast, may offer a more targeted stimulus to the dorsal musculature depending on grip configuration.

In summary, the available evidence indicates that the pullover should not be regarded as a first-choice exercise specifically for maximizing latissimus dorsi EMG amplitude in isolation from the pectoralis major. Its characteristic feature is rather its capacity to engage both the latissimus dorsi and the pectoralis major within a single movement pattern, with shoulder joint position emerging as an important determinant of the relative contribution of each muscle. For practitioners specifically targeting the latissimus dorsi, alternative or complementary pulling exercises may be considered on the basis of the EMG evidence reviewed, while the pullover retains its characteristic value as a multi-muscle movement. It should be noted that the current evidence base is limited to three small studies (combined *n* ≈ 43, all or predominantly male), the exclusive use of isometric protocols in one study [8], and the absence of systematic investigation of technical variants, all of which constrain the generalizability of the conclusions drawn.

### 3.3. Seated Row

The seated row is a compound pulling exercise extensively used in strength and rehabilitation contexts for the development of the posterior kinetic chain, with reference to the latissimus dorsi, trapezius, and posterior deltoid [34]. The available literature on this exercise (five primary studies identified, combined *n* ≈ 81) is characterized by considerable methodological heterogeneity, with studies examining a range of biomechanical variables including grip width, scapular position, shoulder abduction angle, and motor control strategies, under both dynamic and isometric conditions.

Scapular dynamics represent one of the most investigated aspects of seated row execution. In a study of 12 healthy males with at least six months of resistance-training experience, using a self-selected 10–12 RM load, active scapular retraction during the movement did not further increase EMG amplitude of the middle trapezius or rhomboids compared to execution without deliberate retraction, nor did it alter latissimus dorsi EMG amplitude, although the authors noted, on the basis of earlier biomechanical work rather than their own measurements, that this position may contribute to greater glenohumeral joint stability throughout the movement (Lehman et al., 2004 [23]). This evidence derives exclusively from isometric testing conditions, which limits its direct transferability to the dynamic execution typical of resistance training.

Motor control strategies have also been shown to influence EMG amplitude patterns during the seated row, at least transiently. In a study of 20 untrained participants with little or no resistance-training background, the use of mind-muscle connection verbal cues produced an initial increase in latissimus dorsi EMG amplitude accompanied by a concurrent reduction in posterior deltoid EMG amplitude; however, this effect progressively diminished across repetitions and tended to disappear by the final repetitions of the set (Fujita et al., 2020 [24]). This attenuation pattern suggests that attentional-focus strategies may be of limited sustained utility during higher-volume training, at least in individuals with little resistance-training experience; whether the effect differs in trained individuals has not, to our knowledge, been directly tested.

Shoulder abduction angle appears to be a key determinant of EMG amplitude distribution during the seated cable row. In a mixed sample of 11 recreationally trained men and 10 women, higher abduction angles, particularly 60° and 90°, increased EMG amplitude of the upper and middle trapezius and posterior deltoid, whereas lower angles favored latissimus dorsi EMG amplitude under both dynamic and isometric conditions (de Abreu Vasconcelos et al., 2023 [25]). This finding carries a plausible practical implication: practitioners seeking to maximize LD EMG amplitude might favor more adducted arm positions during execution, while wider abduction angles may be more relevant when the training objective targets scapular or posterior-shoulder musculature.

The role of scapular mobility during the movement has been further examined through high-density electromyography in 14 resistance-trained males (age 25 ± 4 years), which revealed broadly similar overall EMG amplitude between fixed-scapula and free-scapula variants across most muscles analyzed. The fixed-scapula variant elicited greater posterior deltoid EMG amplitude during the concentric phase and higher middle trapezius and latissimus dorsi EMG amplitude during the eccentric phase (Padovan et al., 2026 [26]), suggesting that constraining scapular motion may selectively increase EMG amplitude in specific muscles and phases of the movement.

Grip width represents an additional variable with meaningful consequences for the distribution of EMG amplitude. In a separate HD-sEMG study of 14 resistance-trained men, a narrow grip produced consistently greater latissimus dorsi EMG amplitude across both the concentric and eccentric phases (ES = 1.08), whereas a wide grip increased EMG amplitude of the upper trapezius (ES = 1.35 concentric; 2.79 eccentric), middle trapezius (ES = 1.24; 1.44), lower trapezius (ES = 0.90; 0.71), lateral deltoid (ES = 1.03; 0.58) and the erector spinae during the eccentric phase only (ES = 0.65) (Padovan et al., 2025 [9]). This is one of only two studies in the present review to report standardized effect sizes, the other being Fischer et al. (2025 [27]). This pattern, in which narrower and more adducted configurations favor latissimus dorsi EMG amplitude while wider configurations distribute EMG amplitude across scapular stabilizers and accessory musculature, is specific to the seated row and to the manipulation of shoulder-abduction angle: a wider grip was associated with greater latissimus dorsi EMG amplitude in the bent-over row [10], and grip width showed no significant effect in the lat pull-down [1,18]. It should not therefore be generalized as a principle applying across pulling exercises.

However, the strength of this evidence remains constrained by relatively small sample sizes (*n* = 12–21 per study), the predominance of young resistance-trained or untrained participants, one study combining male and female participants without exercise-specific sex-stratified analysis reported here, and considerable methodological heterogeneity across studies, particularly regarding testing protocols (isometric vs. dynamic), exercise execution, and the muscles analyzed.

### 3.4. Barbell Row

The barbell row and its derivatives constitute a broad family of horizontal pulling exercises whose mechanical demands can be substantially modulated through variations in grip configuration, range of motion, body position, and degree of trunk stabilization required. The available evidence across these variants (three primary studies identified) provides a preliminary picture of how execution parameters selectively influence the EMG amplitude of the primary muscles of the posterior upper body; direct cross-study comparison is limited because the three studies used different populations, loads, and testing positions.

Range of motion represents a variable with potentially relevant implications for latissimus dorsi EMG amplitude. In a study of 16 resistance-trained males performing the prone barbell row at 10RM, mean LD excitation was significantly greater in the upper half of the range of motion than in both the lower half (*p* < 0.001, *d* = 0.59) and the full range of motion (*p* < 0.001, *d* = 0.58), suggesting that the initial phases of the pulling movement constitute a window associated with higher target-muscle EMG amplitude (Fischer et al., 2025 [27]). In contrast, the transverse portion of the trapezius exhibited lower peak excitation in the upper half of the range of motion relative to the lower half (*p* = 0.042, *d* = 0.42) and the full range of motion (*p* = 0.013, *d* = 0.54), indicating a dissociation between the EMG amplitude profiles of these two muscle groups across the movement arc. For the posterior deltoid and biceps brachii, no significant differences emerged between range-of-motion conditions [27]. Taken together, these findings, from a single study, suggest that range-of-motion manipulation, when time under tension is held constant, produces moderate and muscle-specific effects on EMG amplitude.

Body position and degree of trunk support exert a more pronounced influence on the distribution of EMG amplitude across the posterior chain. In a study of seven healthy men from a university population, comparing the inverted row, standing bent-over row, and standing one-armed cable row, the inverted row produced the highest LD and upper-back EMG amplitude while simultaneously imposing the lowest lumbar spinal load among the three variants tested (Fenwick, Brown & McGill, 2009 [28]). This remains, to our knowledge, the only study to directly compare these rowing variants within the same sample; given the very small sample size (*n* = 7), this finding, while informative, should be considered preliminary pending replication in a larger cohort before being treated as a definitive practical recommendation.

Grip width and forearm orientation during the incline bench barbell row selectively influenced the EMG amplitude of specific muscle groups without producing global changes in latissimus dorsi EMG amplitude, according to a conference abstract identified in the search (Fusco et al., 2025 [17]). A wide pronated grip was reported to produce significantly greater middle trapezius EMG amplitude compared to narrow pronated, narrow supinated and wide supinated configurations, with no significant differences in latissimus dorsi, upper trapezius or lower trapezius EMG amplitude observed across grip variants. Because this source is a conference abstract rather than a full peer-reviewed article, methodological details including the exact sample size are not reported in full; its findings are therefore presented here as preliminary rather than conclusive.

Interpretation of these findings should consider the limited and heterogeneous sample sizes (*n* = 7–16), the inclusion of predominantly young and recreational or resistance-trained male individuals, the use of a conference abstract as one of three primary sources, and variability in exercise protocols, testing positions, and normalization procedures, all of which limit direct comparison between studies within this exercise family.

### 3.5. Bent-Over Row

The bent-over row is a fundamental compound exercise for the development of the posterior upper-body musculature, primarily targeting the latissimus dorsi, posterior deltoid, and trapezius. Despite its widespread use, the evidence base regarding the influence of execution variables on EMG amplitude remains comparatively limited (two primary studies identified), with available studies focusing primarily on grip width and postural configuration.

With respect to grip width, in a study of 20 active male university students (age 21.1 ± 1.05 years) performing the exercise at 60% 1RM, a wide grip set at 150% of biacromial distance produced greater mean latissimus dorsi EMG amplitude than a narrow grip at 100% of biacromial distance, with no significant interaction effects across sets (Swann & Barry, 2025 [10]). Some intra-subject variability in EMG amplitude was noted between right and left limbs and across repetitions, with a non-linear response pattern particularly evident under wide-grip conditions, indicating that individual biomechanical factors may modulate the EMG response and that the effect of grip width cannot be assumed uniform across all practitioners; this finding derives from a single study in young trained men and has not yet been replicated.

Postural configuration represents a further variable with implications for EMG amplitude and estimated spinal loading. In a preliminary study of three young resistance-trained university students, bench-supported variants, which eliminate the need for active trunk stabilization, showed lower lumbar and thoracic spinal erector EMG amplitude than standing execution, although none of the between-condition comparisons reached statistical significance (García-Jaén et al., 2021 [29]). While this may be relevant in contexts where spinal load management is a priority, it corresponds to reduced global trunk-extensor EMG amplitude as well. Regarding upper-back musculature, shoulder abduction angle played a role in determining which muscles were preferentially recruited: greater shoulder abduction, particularly in bench-supported conditions, favored posterior deltoid and upper trapezius EMG amplitude, whereas latissimus dorsi EMG amplitude was comparatively reduced in this configuration [29]. Conversely, standing execution maintained higher trunk-extensor EMG amplitude while preserving EMG amplitude of the primary pulling muscles. Because the source explicitly identifies itself as preliminary, includes only three participants and reports no statistically significant between-condition differences, these observations are reported as directional trends and are not advanced as a basis for a practical recommendation.

Nevertheless, the interpretation of these findings is limited by very small sample sizes, preliminary study designs, and differences in experimental procedures, which reduce the robustness and external validity of the conclusions.

### 3.6. Pull-Ups

Pull-ups and chin-ups represent the closed-chain counterpart of the lat pull-down and are widely used exercises for developing upper-body pulling strength. Although the two movements share a broadly similar mechanical structure, grip orientation and width introduce differences in the relative EMG amplitude of the muscles involved, with potential implications for exercise selection and program design; this exercise family is supported by four primary studies, combined *n* ≈ 84.

The most consistently reported distinction in the literature concerns the comparison between pronated (pull-up) and supinated (chin-up) grip orientations. In a mixed sample of 21 men and 4 women, supinated grips elicited greater biceps brachii and pectoralis major EMG amplitude, whereas pronated grips were associated with comparatively higher lower trapezius EMG amplitude (Youdas et al., 2010 [30]). This differential pattern reflects the biomechanical influence of forearm orientation on elbow-flexor mechanics and glenohumeral positioning and provides a rationale for selecting one variant over the other depending on the target musculature. A further observation of practical relevance concerns the temporal sequence of EMG amplitude throughout the movement: scapular muscles were recruited predominantly in the initial phase, with biceps brachii and latissimus dorsi EMG amplitude increasing progressively toward the terminal concentric phase [30], suggesting that the neuromuscular demand is not uniformly distributed across the range of motion.

With respect to grip width, in a study of 10 healthy men performing six grip variations, wider pronated grips were associated with a slight reduction in latissimus dorsi EMG amplitude, while biceps brachii EMG amplitude remained more sensitive to grip orientation than to grip width per se (Raizada & Bagchi, 2019 [31]). Across different pull-up variants including supinated, pronated, neutral and rope-based grips, in a study of 19 strength-trained males (age 24.9 ± 5 years), mean and peak EMG amplitude of the primary shoulder, arm and forearm muscle complex appeared broadly comparable in magnitude, with differences emerging primarily in the temporal distribution of EMG amplitude between concentric and eccentric phases rather than in overall EMG amplitude (Dickie et al., 2017 [32]). This suggests that grip variation modifies the timing and relative contribution of individual muscles more than it alters the total neuromuscular demand of the exercise.

The use of grip-assistance devices, such as Versa Grips, in a study of 30 healthy strength-trained males, selectively reduced wrist flexor and extensor EMG amplitude without meaningfully altering latissimus dorsi or infraspinatus EMG amplitude (Escalante et al., 2015 [33]). This finding implies that grip modifications primarily affect forearm musculature and may be relevant in managing localized fatigue or injury risk at the wrist and forearm, without compromising the EMG amplitude delivered to the primary pulling muscles.

## 4. Discussion

In relation to the stated objective of this review—identifying which exercises and technical variations are most consistently associated with higher latissimus dorsi EMG amplitude—the synthesis in Section 3 supports a within-family rather than a single cross-exercise answer. Within the lat pull-down family, the anterior pull direction is the most consistently supported configuration: it was associated with a more distributed EMG amplitude profile and greater concentric LD amplitude than the behind-the-neck variant [19], and no study reported an LD amplitude advantage for the posterior variant outside the eccentric phase of a single HD-sEMG comparison, which conversely favoured the anterior variant in the concentric phase [20]. Grip width, by contrast, produced inconsistent findings: an advantage for the wide grip was reported in one early study of ten participants [15], but no significant effect was found in the two larger studies [1,18]; grip width is therefore not advanced here as a determinant of LD EMG amplitude. Forearm orientation was examined in a single study, which reported greater LD amplitude with a pronated than with a supinated grip [14]; this remains unreplicated. Within the seated row family, two separate execution variables are each individually associated with higher LD EMG amplitude: a narrower grip, shown in an HD-sEMG study with one of the highest methodological-quality indicators in this review (score 6/6, tied with two other HD-sEMG studies; Table 2) [9], and a lower shoulder-abduction angle, shown in a separate conventional-sEMG study [25]. These two variables have not, to our knowledge, been tested in combination within a single study, so a narrow grip combined with a low abduction angle should be read as two independently supported observations rather than a jointly validated configuration; this distinction is noted explicitly in Table 3. Among rowing variants more broadly, the inverted row was associated with the highest LD EMG amplitude in the only study directly comparing rowing exercises within the same sample [28], although, as noted in Section 3.4, this rests on a very small sample and should be treated as preliminary. Pull-up and chin-up variants show broadly comparable LD EMG amplitude regardless of grip, so that grip selection within this family can reasonably be guided by secondary goals such as elbow-flexor emphasis rather than by LD amplitude itself [30,31,32]. The pullover, by contrast, does not emerge as a first-choice LD-selective exercise, since its EMG amplitude profile is more heavily weighted toward the pectoralis major and is strongly dependent on shoulder joint angle rather than on exercise selection per se [8,21,22]. A single, statistically valid ranking across exercise families is not supported by the current evidence, because the included studies differ in normalization baseline, population and testing conditions in ways that preclude direct amplitude comparison across different exercises tested in different samples; this constraint, rather than a gap in the analysis, is the reason the synthesis above is presented at the within-family level. These within-family patterns are broadly consistent with general biomechanical principles described in the wider strength and conditioning literature regarding the influence of joint angle and lever arm on relative muscle contribution during multi-joint pulling movements [37], although a systematic comparison against that broader literature was outside the scope of the present search.

The available evidence indicates that latissimus dorsi EMG amplitude during pulling exercises is, in most of the comparisons reviewed, only marginally influenced by the principal execution variables tested (grip width, forearm orientation), whereas biomechanical modifications more consistently redistribute EMG amplitude among synergistic and stabilizing muscles. Exercise-specific variations in grip, shoulder position, trunk inclination, and body support appear to modulate the neuromuscular recruitment strategy more consistently than they alter latissimus dorsi EMG amplitude itself. This pattern is drawn primarily from between-exercise and between-study comparisons using non-identical loads, normalization procedures, and electrode configurations, and should therefore be regarded as a provisional synthesis rather than a firmly established principle. With this caveat, exercise selection may reasonably be guided by secondary programming objectives—regional muscular emphasis, spinal loading, joint positioning, fatigue management, and individual training goals—in addition to, rather than instead of, considerations of latissimus dorsi EMG amplitude.

It is important to distinguish clearly between the findings summarized above and claims about training effectiveness. Acute surface EMG amplitude reflects the level of muscle excitation during a single testing session and is, at best, an indirect and imperfect proxy for the mechanical and metabolic stimuli that drive hypertrophic or strength adaptations over weeks to months of training. None of the studies reviewed here included a longitudinal training intervention, and none reported hypertrophy, strength, or functional-performance outcomes. Consequently, terms such as “effectiveness,” “optimal,” or “best” exercise, when they appear in the literature discussed above, should be understood as referring to acute EMG amplitude and not to demonstrated superiority for muscle growth or long-term performance.

Several further limitations warrant explicit discussion. First, the reviewed studies differ substantially in relative load, repetition number, movement velocity, fatigue level, range of motion, contraction type (isometric vs. dynamic), electrode placement, signal processing, and normalization procedure; each of these factors can materially alter measured EMG amplitude independently of the biomechanical variable nominally under study. Furthermore, direct comparisons between exercises are scarce, while high-quality studies systematically evaluating multiple technical variables within standardized experimental designs remain limited. Direct ranking of exercises across studies that differ on several of these dimensions simultaneously is not statistically justified, and the exercise-level summaries in Table 3 should be read as qualitative, hypothesis-generating syntheses rather than as evidence-graded rankings in the systematic-review sense.

Second, surface EMG has recognized anatomical and technical limitations for a muscle as large as the latissimus dorsi. Conventional bipolar sEMG electrodes sample a relatively small, fixed region of the muscle and may therefore preferentially represent specific portions of its broad, fan-shaped architecture, potentially missing regional variation in excitation across its origin-to-insertion span. Cross-talk from adjacent musculature (e.g., teres major, lower trapezius, and the thoracolumbar paraspinals) and inter-individual differences in subcutaneous adipose tissue thickness can further influence signal amplitude independently of true muscle excitation. The HD-sEMG studies included in this review (Padovan et al., 2024 [20]; 2025 [9]; 2026 [26]) partially address this limitation by mapping excitation across a grid of electrodes rather than a single site, and their findings of phase- and region-specific excitation differences (e.g., greater eccentric-phase LD excitation with a fixed scapula [26]) illustrate that conventional single-site sEMG may under- or over-represent whole-muscle behavior. Where conclusions in this review rely on conventional sEMG alone, this limitation should be borne in mind, and comparisons between conventional-sEMG and HD-sEMG studies should be made cautiously given their different spatial resolution.

Third, several of the practical observations summarized in Table 3 rest on a single study, a small and often exclusively male sample, or, in one instance, a conference abstract that does not report its methodological details in full (Fusco et al., 2025 [17]). These findings are presented as preliminary and are explicitly distinguished in Table 3 from patterns supported by multiple, directionally consistent studies (e.g., the broad comparability of LD EMG amplitude across pull-up grip variants, replicated across four studies). Safety-related observations, in particular regarding the behind-the-neck lat pull-down and the inverted row, are grounded in plausible biomechanical reasoning about joint position and estimated spinal load rather than in studies that directly measured injuries, symptoms, or prospective adverse outcomes; they are framed accordingly throughout this review as biomechanical considerations rather than demonstrated differences in injury risk.

Regarding the relationship between humerus-to-trunk position and load distribution among pulling muscles, discussed in the Conclusions, this pattern emerges from qualitative comparison across the exercise-specific findings summarized in Section 3.1, Section 3.2, Section 3.3, Section 3.4, Section 3.5 and Section 3.6 (e.g., the shoulder-angle dependence of pectoralis major versus latissimus dorsi excitation in the pullover [8], and the shoulder-abduction-angle dependence of latissimus dorsi versus trapezius excitation in the seated row [25]) rather than from a single study directly designed to test this hypothesis across exercises within the same sample. It is best understood as a biomechanically plausible inference drawn across studies rather than a directly demonstrated finding, and we present it here as a candidate organizing principle for future hypothesis-driven research rather than as an established conclusion.

Taken together, the findings that are supported by multiple, directionally consistent studies include: the relative insensitivity of latissimus dorsi EMG amplitude to grip width in the lat pull-down (three studies) and in pull-up variants (four studies); and the shoulder-position dependence of relative muscle contribution in both the pullover and the seated row (each supported by more than one study, albeit with different designs). Findings resting on a single study—including the inverted row comparison, the range-of-motion effect in the barbell row, the bent-over row postural comparison, and the incline-row grip finding—are noted as such and should be treated as preliminary. No finding in this review, however well replicated at the level of acute EMG amplitude, should be interpreted as establishing the long-term training superiority of one exercise over another; this would require dedicated longitudinal resistance-training studies with hypertrophy, strength, or performance outcomes, which are currently absent from this literature.

Future research should prioritize larger and more diverse cohorts (including female and older adult populations, which are essentially absent from the reviewed evidence base), standardized EMG methodologies with harmonized normalization procedures, wider use of HD-sEMG to address the regional-activation limitations of conventional sEMG, and, most importantly, longitudinal training studies that connect acute EMG amplitude findings to actual hypertrophic and strength outcomes. Such investigations are required to move from the current, largely descriptive and exploratory evidence base toward robust, causally grounded recommendations for exercise prescription.

## 5. Conclusions

In direct response to the objective of this review, the exercise characteristics most consistently associated with greater latissimus dorsi EMG amplitude within the available evidence are as follows. Within the lat pull-down family, the anterior pull direction is supported by the broadest evidence base among the configurations reviewed, whereas grip width did not produce a consistent effect on latissimus dorsi EMG amplitude and is therefore not advanced as a determinant. Within the seated row family, a narrower grip and a lower shoulder-abduction angle were each associated with greater latissimus dorsi EMG amplitude in separate studies; these should be considered independently supported variables, as their combination has not, to our knowledge, been directly tested within a single study. Among rowing exercises more broadly, the inverted row showed the highest latissimus dorsi EMG amplitude in the only study directly comparing rowing variants within the same sample. However, this finding should be regarded as preliminary because it derives from a single study involving only seven participants and scored in the lowest band of our methodological-quality indicator (Table 2). It is therefore not advanced with the same level of confidence as the findings above. Pull-up and chin-up variants showed broadly comparable latissimus dorsi EMG amplitude across grip configurations, while the pullover is better characterized as a combined latissimus dorsi–pectoralis major movement than as a latissimus-dorsi-selective exercise. Because the included studies differed in normalization procedures, participant characteristics, loading conditions, and testing protocols, these observations are best interpreted within individual exercise families rather than as a single ranking of exercises across the entire literature.

Across exercise families, shoulder and humeral position relative to the trunk was consistently associated with shifts in the relative contribution of the latissimus dorsi and synergistic muscles, including the pectoralis major during the pullover and the trapezius and posterior deltoid during the seated row. This pattern suggests that shoulder and humeral position may represent a candidate unifying biomechanical determinant of latissimus dorsi involvement across exercises. However, this interpretation is based on inference across studies rather than on direct within-study comparisons and should therefore be considered hypothesis-generating rather than definitive (Section 4).

These conclusions should be interpreted in light of several important limitations. First, several exercise-specific observations—including the inverted row’s favorable EMG-amplitude-to-estimated-spinal-load profile and the behind-the-neck pull-down’s comparatively unfavorable biomechanical position—are derived from a single or very small study (Table 2) and require replication before they can be considered firm, generalizable findings; this is indicated explicitly for each exercise in Table 3. Second, and more fundamentally, acute EMG amplitude provides an indirect measure of neuromuscular demand during a single testing session and cannot, by itself, determine which exercise produces superior long-term hypertrophy, strength, or functional outcomes, as none of the studies reviewed included a longitudinal training intervention. Methodological limitations common to this literature—including small and predominantly male samples, heterogeneous loading and normalization protocols, near-exclusive reliance on conventional surface EMG, and the near-total absence of longitudinal training and injury-outcome data—further limit the strength and generalizability of the available evidence.

Accordingly, the findings of this review should be considered the best currently available evidence-informed basis for exercise selection rather than a definitive or evidence-graded prescription of a single optimal or first-choice exercise. Strength and conditioning professionals should therefore consider these findings alongside individual training goals, exercise tolerance, and practical constraints, while recognizing that longitudinal training evidence is currently insufficient to establish whether the acute EMG differences identified between exercise configurations translate into superior long-term adaptations. Future research should prioritize longitudinal training interventions, injury-related outcomes, and adequately powered comparisons across diverse populations, as these represent important gaps in the current literature (Section 4).

## Figures and Tables

**Table 1:** Synthesis of exercise-specific biomechanical variables and their reported effects on latissimus dorsi and synergistic muscle EMG amplitude across the pulling exercises reviewed, with methodological characteristics of each included study. Effects are reported as described in the corresponding primary studies.

Exercise	Study	Sample (*n*; Sex; Training Status)	Load/Protocol	Assessment	EMG Normalization/Electrodes	Biomechanical Variable	Effect on LD EMG Amplitude	Effect on Synergistic/Stabilizing Muscles
Lat Pull-down	Signorile et al., 2002 [15]	*n* = 10; men; healthy	10RM, 3 reps, 4 hand positions	Dynamic	sEMG, %MVIC (NR detail)	Grip width/hand position	Wide anterior grip > narrow/supinated (concentric & eccentric)	NR
	Andersen et al., 2014 [18]	*n* = 15; men; NR training status	6RM, narrow/medium/wide grip	Dynamic	sEMG, %MVIC	Grip width	No significant difference across grip widths	Medium/narrow grip associated with higher biceps brachii amplitude
	Buonsenso et al., 2025 [1]	*n* = 40; men; ≥5 y resistance-trained	70% 1RM, 5 reps	Dynamic	sEMG, %MVIC	Grip width; trunk inclination (~30° posterior lean + wide pronated grip)	No significant difference across grip widths; no change in LD with trunk inclination	Significant increase in posterior deltoid amplitude with trunk inclination only
	Lusk et al., 2010 [14]	*n* = 12; men; healthy	70% 1RM, 4 grip/orientation variants	Dynamic	sEMG, %MVIC	Forearm orientation (pronated vs. supinated)	Pronated > supinated	Supinated grip: greater biceps brachii amplitude
	Sperandei et al., 2009 [19]	*n* = 24; men; trained adults	80% 1RM, 5 reps; BNL/FNL/V-bar	Dynamic	sEMG, %MVIC	Pull direction (anterior vs. posterior)	Anterior: greater concentric LD amplitude	Anterior: greater pectoralis major; Posterior: greater posterior deltoid/biceps brachii
	Padovan et al., 2024 [20]	*n* = 14; men; resistance-trained	8RM, front vs. back pull-down	Dynamic	HD-sEMG	Pull direction (front vs. behind-the-neck)	Anterior > posterior in the concentric phase; posterior > anterior in the eccentric phase	Muscle-specific spatial excitation differences (HD-sEMG mapping)
Pull-over	Marchetti & Uchida, 2011 [21]	*n* = 8; men; healthy volunteers	30% body weight, 10 reps	Dynamic (concentric/eccentric)	sEMG, %MVIC	Pullover execution	Lower amplitude than pectoralis major	Pectoralis major more heavily emphasized
	Teixeira et al., 2022 [8]	*n* = 15; men; recreationally trained	Isometric, shoulder angle 0–180°	Isometric	sEMG, %MVIC	Shoulder joint angle; exercise choice (pullover vs. pull-down)	LD/posterior deltoid patterns comparable between exercises	Pectoralis major amplitude highly sensitive to joint angle
	Muyor et al., 2022 [22]	*n* = 20; sex NR; healthy, physically active	30% body weight, 8 reps	Dynamic	sEMG, %MVIC	Pullover vs. straight-arm pulldown, grip width	LD not increased in pullover (exception, with triceps brachii)	Pullover elicits higher amplitude in most other muscles studied
Seated Row	Lehman et al., 2004 [23]	*n* = 12; men; healthy	Scapular retraction (active vs. passive)	Isometric	sEMG, %MVIC	Scapular retraction	No change with active vs. passive scapular retraction	No further increase in middle trapezius/rhomboid amplitude with active retraction
	Fujita et al., 2020 [24]	*n* = 20; sex NR; untrained	Mind–muscle cueing, to failure	Dynamic	sEMG, %MVIC	Verbal cueing (attentional focus)	Transient increase, diminishing across reps	Concurrent transient reduction in posterior deltoid amplitude
	de Abreu Vasconcelos et al., 2023 [25]	*n* = 21 (11 men, 10 women); recreationally trained	Shoulder abduction 30°/60°/90°	Dynamic & isometric	sEMG, %MVIC	Shoulder abduction angle	Lower angles favor LD amplitude	Higher angles (60°, 90°) increase upper/middle trapezius, posterior deltoid
	Padovan et al., 2026 [26]	*n* = 14; men (25 ± 4 y); resistance-trained	8RM, fixed vs. free scapula	Dynamic	HD-sEMG	Scapular mobility	Greater LD amplitude, fixed-scapula, eccentric phase	Fixed-scapula: greater posterior deltoid (concentric), middle trapezius (eccentric)
	Padovan et al., 2025 [9]	*n* = 14; men; resistance-trained	8RM, narrow vs. wide grip	Dynamic	HD-sEMG	Grip width	Narrow grip > wide grip (both phases)	Wide grip increases upper/middle/lower trapezius, lateral deltoid
Barbell Row	Fischer et al., 2025 [27]	*n* = 16; men; resistance-trained	10RM, prone row, ROM upper/lower/full	Dynamic	sEMG, %MVIC	Range of motion	Upper-half ROM > lower-half (*d* = 0.59) and full ROM (*d* = 0.58)	Transverse trapezius lower peak excitation in upper half; posterior deltoid/biceps brachii unaffected
	Fenwick, Brown & McGill, 2009 [28]	*n* = 7; men; university population	Inverted row vs. standing bent-over row vs. 1-arm cable row	Dynamic	sEMG (NR normalization detail)	Body position/trunk support	Inverted row highest among 3 variants	Inverted row: highest upper-back amplitude; lowest lumbar spinal load
	Fusco et al., 2025 [17]—conference abstract, preliminary	*n* NR	Incline bench row, grip width/orientation	Dynamic	NR	Grip width/orientation	No significant differences across grip variants	Wide pronated grip: greater middle trapezius amplitude only
Bent-Over Row	Swann & Barry, 2025 [10]	*n* = 20; men (21.1 ± 1.05 y); active university students	60% 1RM, 100% vs. 150% biacromial grip	Dynamic	sEMG, %MVIC	Grip width	Wide grip (150%) > narrow grip (100%)	NR; notable inter-limb/inter-repetition variability
	García-Jaén et al., 2021 [29]—preliminary study	*n* = 3; young men; resistance-trained university students	Standing vs. bench-supported	Dynamic	sEMG, %MVIC	Postural configuration	Reduced with greater abduction, bench-supported (not statistically significant)	Bench: reduced lumbar/thoracic erector amplitude, greater posterior deltoid/upper trapezius at high abduction
Pull-ups/Chin-ups	Youdas et al., 2010 [30]	*n* = 25 (21 men, 4 women); healthy	Pull-up/chin-up/Perfect-Pullup	Dynamic	sEMG, %MVIC	Grip orientation; temporal pattern	Increases toward terminal concentric phase	Supinated: greater biceps brachii/pectoralis major; Pronated: greater lower trapezius
	Raizada & Bagchi, 2019 [31]	*n* = 10; men; healthy	1 rep, 6 grip variations	Dynamic	sEMG, %MVIC	Grip width	Slight reduction with wider pronated grips	Biceps brachii more sensitive to orientation than width
	Dickie et al., 2017 [32]	*n* = 19; men (24.9 ± 5 y); strength-trained	Supinated/pronated/neutral/rope	Dynamic	sEMG, %MVIC	Grip variant	Broadly comparable mean/peak amplitude	Differences in concentric/eccentric temporal distribution only
	Escalante et al., 2015 [33]	*n* = 30; men; strength-trained	Versa Grips vs. standard grip	Dynamic	sEMG, %MVIC	Grip-assistance device	No meaningful alteration	Selective reduction in wrist flexor/extensor amplitude

Abbreviations: LD = latissimus dorsi; NR = not reported by the primary source; RM = repetition maximum; sEMG = surface electromyography; HD-sEMG = high-density surface electromyography; %MVIC = percentage of maximal voluntary isometric contraction; Electrode location is not tabulated study-by-study because it is not uniformly reported across the included studies; where it is described, placement was broadly consistent with SENIAM recommendations, as discussed at the beginning of Section 3.

**Table 2:** Methodological-quality indicator for each included primary study, based on the transparent six-criterion checklist described in Section 2.5 (informed by Downs & Black, 1998 [35], and SENIAM recommendations [36]). This is a review-specific descriptive tool, not a validated risk-of-bias instrument.

Study	C1: *n* ≥ 12	C2: Sex & Training Status Reported	C3: Load Standardized	C4: EMG Normalization Reported	C5: Dynamic Assessment	C6: HD-sEMG	Score (/6)
Signorile et al., 2002 [15]	0	1	1	1	1	0	4
Andersen et al., 2014 [18]	1	0	1	1	1	0	4
Buonsenso et al., 2025 [1]	1	1	1	1	1	0	5
Lusk et al., 2010 [14]	1	1	1	1	1	0	5
Sperandei et al., 2009 [19]	1	1	1	1	1	0	5
Padovan et al., 2024 [20]	1	1	1	1	1	1	6
Marchetti & Uchida, 2011 [21]	0	1	1	1	1	0	4
Teixeira et al., 2022 [8]	1	1	1	1	0	0	4
Muyor et al., 2022 [22]	1	0	1	1	1	0	4
Lehman et al., 2004 [23]	1	1	0	1	0	0	3
Fujita et al., 2020 [24]	1	0	0	1	1	0	3
de Abreu Vasconcelos et al., 2023 [25]	1	1	0	1	1	0	4
Padovan et al., 2026 [26]	1	1	1	1	1	1	6
Padovan et al., 2025 [9]	1	1	1	1	1	1	6
Fischer et al., 2025 [27]	1	1	1	1	1	0	5
Fenwick, Brown & McGill, 2009 [28]	0	1	0	0	1	0	2
Fusco et al., 2025 [17]—conference abstract	0	0	0	0	1	0	1
Swann & Barry, 2025 [10]	1	1	1	1	1	0	5
García-Jaén et al., 2021 [29]—preliminary study	0	0	0	1	1	0	2
Youdas et al., 2010 [30]	1	1	1	1	1	0	5
Raizada & Bagchi, 2019 [31]	0	1	1	1	1	0	4
Dickie et al., 2017 [32]	1	1	1	1	1	0	5
Escalante et al., 2015 [33]	1	1	1	1	1	0	5

Abbreviations: HD-sEMG = high-density surface electromyography. Score bands (descriptive only, not a validated cut-off): 5–6 = higher; 3–4 = moderate; 0–2 = lower. Three studies score in the “lower” band (Fenwick, Brown & McGill, 2009 [28]; Fusco et al., 2025 [17]; García-Jaén et al., 2021 [29]); consistent with Section 2.5, their findings are treated as preliminary throughout Section 3 and Section 4 and are not the sole basis for any practical recommendation.

**Table 3:** Practical synthesis of the exercise configurations most consistently associated with greater latissimus dorsi EMG amplitude in the reviewed literature, together with the principal biomechanical consideration, the certainty of the underlying evidence, and the main methodological limitation reported for each exercise category. These are exercise-selection considerations grounded in acute EMG amplitude data; they are not claims of superior long-term hypertrophic, strength, or safety outcomes, which would require dedicated longitudinal and injury-surveillance research.

Exercise	Configuration Associated with Greater LD EMG Amplitude	Key Biomechanical Consideration	Certainty of Evidence	Primary Reported Limitation
Lat Pull-down	Anterior pull direction. Grip width is not supported as a determinant of LD EMG amplitude	The posterior (behind-the-neck) variant shows no consistent LD amplitude advantage and is associated, on biomechanical grounds only, with greater theoretical cervical-spine/shoulder-joint stress; not verified against injury outcomes	Moderate for pull direction—anterior direction favored in 1 conventional-sEMG study [19], with a concentric-phase advantage for the anterior variant and an eccentric-phase advantage for the posterior variant in the HD-sEMG comparison [20]. Inconsistent for grip width—no significant effect in the 2 larger studies [1,18], against 1 smaller and earlier study reporting an advantage for the wide grip [15]. Pronated vs. supinated orientation: single unreplicated study [14]	Small-to-moderate, exclusively-male samples; near-exclusive reliance on surface EMG
Pull-over	Not a first-choice exercise for LD-selective EMG amplitude; useful for combined LD–pectoralis major stimulus	Shoulder joint angle, more than exercise choice, shifts emphasis between LD and pectoralis major	Low—based on 3 small studies (combined *n* ≈ 43), one isometric-only	Small, predominantly single-sex samples; isometric-only protocol in one study [8]; no systematic testing of technical variants
Seated Row	Narrow grip and low shoulder-abduction angle (two independently-supported variables, not yet tested in combination in a single study)	Mind–muscle-connection cueing may increase LD amplitude only transiently, mainly reported in untrained individuals [24]	Moderate—grip-width finding from a single HD-sEMG study [9] (score 6/6 in Table 2, tied for the highest score with two other HD-sEMG studies); abduction-angle finding from a separate conventional-sEMG study [25] (score 4/6); both directionally consistent with the broader “narrow/adducted favors LD” pattern seen elsewhere, but not jointly validated	Small samples (*n* = 12–21); predominance of young resistance-trained participants; mix of isometric and dynamic protocols
Barbell Row	Inverted row variant; emphasis on the upper portion of the pulling range of motion	Grip width/orientation changes show little effect on LD amplitude within this family (based on a conference abstract)	Low—inverted-row finding from a single study with *n* = 7; ROM finding from a single study with *n* = 16; grip finding from an unreplicated conference abstract	Very small samples (*n* = 7–16); one source is a conference abstract with unverifiable methodological detail
Bent-Over Row	Wide grip (~150% of biacromial distance), from a single study [10]. No statistically supported configuration identified for trunk support or shoulder position	Bench support was associated with lower spinal erector amplitude but also with lower overall trunk-extensor stimulus; the only available comparison is preliminary and is not a basis for a recommendation	Low—the grip-width finding rests on a single study [10]; the postural comparison [29] comes from a three-participant source explicitly labeled preliminary by its own authors, reported no significant between-condition differences, and is reported as a directional trend only	Very small samples (*n* = 3 and *n* = 20); preliminary study design; procedural heterogeneity
Pull-ups/Chin-ups	Grip orientation and width can be selected based on secondary goals (e.g., elbow-flexor emphasis), since LD amplitude is broadly comparable across variants	Assistance devices (e.g., Versa Grips) can offload forearm musculature without altering the LD stimulus	Moderate—pattern of broad comparability across grip variants replicated across 4 studies (combined *n* ≈ 84), though all-male or male-predominant	General review limitations apply (see Section 4); largely male samples

## Data Availability

No new data were created or analyzed in this study. Data sharing is not applicable to this article.

## Abbreviations

The following abbreviations are used in this manuscript:

HD-EMG	High-Density Electromyography
sEMG	Surface Electromyography
%MVIC	percentage of maximal voluntary isometric contraction
LD	Latissimus dorsi
RM	Repetition maximum
ROM	Range of motion
